# Investigation of the Thermo-Mechanical Properties of a 3D-Printed Carbon Fiber-Reinforced PPA Composite

**DOI:** 10.3390/polym18121422

**Published:** 2026-06-07

**Authors:** Urte Cigane, Tomas Kalinauskis, Justas Ciganas

**Affiliations:** Siauliai State Higher Education Institution, Ausros al. 40, LT 76241 Siauliai, Lithuania; t.kalinauskis@svako.lt

**Keywords:** PPA CF15, quasi-static tensile, fatigue, dynamic mechanical analysis (DMA), additive manufacturing (AM), finite element analysis (FEA), automotive components

## Abstract

This study investigates the thermo-mechanical performance of fused filament fabrication (FFF)-printed polyphthalamide reinforced with 15 wt.% short carbon fibers (PPA CF15) for engineering applications under elevated temperature and cyclic loading conditions. The material was characterized by quasi-static tensile testing, fatigue testing, dynamic mechanical analysis (DMA), scanning electron microscopy (SEM), and finite element analysis (FEA). Tensile tests performed from 20 to 180 °C revealed a strong temperature-dependent reduction in mechanical properties: the elastic modulus decreased from 2.437 to 0.401 GPa, while the ultimate tensile strength decreased from 64.537 to 9.190 MPa. In contrast, elongation at break generally increased with temperature, indicating a transition toward more ductile deformation governed by thermal softening of the polymer matrix. Fatigue tests showed reduced fatigue resistance at higher temperatures and stress levels; however, stable cyclic performance was achieved when the applied stress remained below approximately 60–70% of the ultimate tensile strength, with several specimens reaching 10^6^ cycles. DMA confirmed the viscoelastic nature of PPA CF15 and enabled the construction of frequency–temperature superposition master curves for numerical modelling. SEM observations revealed increased matrix deformation and fiber pull-out at elevated temperatures. FEA of an automotive intake manifold (IM) case study demonstrated that experimentally derived material data can be used to predict deformation, stress redistribution, and viscoelastic stabilization under combined thermal and mechanical loading. The results indicate that FFF-printed PPA CF15 is a promising lightweight composite for thermally and mechanically demanding automotive applications.

## 1. Introduction

Additive manufacturing (AM) technologies have undergone substantial advancement in recent years and are increasingly being implemented for the production of functional engineering components across a broad range of industrial sectors [1,2]. Among the different AM techniques, fused filament fabrication (FFF) has become one of the most widely used polymer-based AM methods due to its relative ease of use, process flexibility, ability to produce geometrically complex structures, and broad adoption in industrial applications [3]. Moreover, AM can provide environmental advantages for low- to medium-volume production by allowing part consolidation, reducing assembly requirements, and eliminating tooling, thus reducing energy use, material waste, and emissions, particularly for complex or lightweight components [4]. Consequently, FFF is increasingly being explored not only for prototyping but also for the production of functional tools and end-use parts [2,5].

Despite the advantages, the broader industrial adoption of FFF is still limited by several material-related challenges [6]. The mechanical performance, thermal resistance, and long-term durability of the printed components are highly dependent on the material system used and the interlayer bonding formed during the printing process [7,8,9,10,11,12,13]. Polymers commonly used in FFF, such as polylactic acid (PLA) [14,15,16,17] and acrylonitrile butadiene styrene (ABS) [18,19,20], exhibit relatively low thermal resistance and limited mechanical performance, restricting their use in demanding engineering environments [21]. Therefore, significant research efforts have been directed toward the development and investigation of high-performance thermoplastics suitable for AM applications [22,23,24,25,26].

Among advanced engineering thermoplastics, polyphthalamide (PPA) has attracted considerable attention due to its combination of high mechanical strength, excellent thermal stability, and superior chemical resistance [27]. PPA is a semi-aromatic polyamide characterized by a relatively high glass transition temperature (*T_g_*), low moisture absorption compared with conventional polyamides, and improved dimensional stability under elevated temperature conditions [28]. These properties make PPA particularly suitable for components operating in harsh environments, including high-temperature mechanical systems and chemically aggressive conditions [29]. Consequently, PPA has been adopted in industries such as electronics [30] and automotive engineering [31,32,33] for the production of structurally demanding components.

To further enhance the performance of PPA-based materials, high-performance fiber reinforcement is used [27]. In particular, carbon fiber (CF) reinforced thermoplastic composites have become increasingly important in modern engineering applications [34,35,36]. Incorporation of carbon fibers into polymer matrices significantly improves stiffness, strength, thermal conductivity, and dimensional stability, while also reducing the coefficient of thermal expansion [37,38,39,40]. These improvements make carbon fiber reinforced polymers attractive candidates for lightweight structural components where high mechanical performance and thermal resistance are required [41,42,43]. As a result, carbon fiber reinforced thermoplastic composites are increasingly being explored in AM technologies, including FFF [44,45,46].

The increasing pressure to improve environmental sustainability, energy efficiency, and overall performance is changing the automotive industry [47,48]. Increasingly strict regulations on fuel consumption and emissions are accelerating the search for innovative materials and engineering solutions that can reduce vehicle mass while ensuring adequate structural strength [49]. Lightweight composite materials, generally composed of a fiber- or particle-reinforced matrix, have emerged as a key technology in this field due to their high strength-to-weight ratios, design versatility and multifunctional capabilities, allowing weight reductions of up to 50% relative to traditional metal components [50]. The integration of composite materials in automotive engineering represents not just a passing trend but a fundamental shift that has the potential to transform vehicle design and manufacturing [51].

The intake manifold (IM) is a key component of the internal combustion engine air management system, playing a crucial role in the regulation of the airflow to the cylinders [52]. The primary function of the IM is to evenly distribute air between the engine cylinders, ensuring efficient combustion and optimal engine performance [53,54]. In modern turbocharged engines, the IM is subjected to demanding thermo-mechanical conditions associated with elevated temperature and pressure levels [55]. Depending on engine configuration and operating regime, intake air temperatures can increase significantly, while pressure fluctuations occur due to dynamic engine operation and turbocharger behavior [56,57].

The demanding thermo-mechanical conditions of modern turbocharged engines impose strict requirements on materials used for IM components, which must maintain mechanical integrity, dimensional stability, and airtight sealing under elevated temperature, pressure, and cyclic load [58,59]. Although IM has traditionally been manufactured from metals such as aluminum, current automotive design increasingly favors lightweight polymer-based solutions due to their lower density, manufacturing flexibility, and potential cost reduction [60,61]. Among these materials, carbon fiber reinforced PPA composites have emerged as promising candidates due to their high stiffness, thermal stability, and reduced thermal deformation [29,62]. In parallel, AM technologies such as FFF enable the production of complex IM geometries with optimized flow paths and lightweight structures. Nevertheless, despite growing research interest in additively manufactured high-performance polymer composites [63,64,65,66], the thermo-mechanical performance of carbon fiber reinforced PPA fabricated through FFF has not yet been comprehensively investigated. Moreover, systematic investigations of the thermo-mechanical and fatigue behavior of FFF-printed PPA CF15 composites over a broad temperature range remain limited. In particular, the combined evaluation of quasi-static tensile properties, fatigue performance, DMA characterization, and thermo-mechanical numerical modelling under elevated temperatures has not yet been comprehensively reported [67].

Therefore, this study investigates the thermo-mechanical performance of PPA reinforced with 15 wt.% short carbon fibers (PPA CF15) produced using FFF technology. The experimental program encompassed quasi-static tensile testing, fatigue characterization, and dynamic mechanical analysis (DMA), all performed under rigorously controlled thermal conditions. DMA was conducted to characterize the temperature-dependent viscoelastic behavior of the material. In addition, scanning electron microscopy (SEM) was performed to examine fatigue-related damage mechanisms. The obtained experimental data were further applied in analytical calculations to assess the structural response of representative geometries under combined thermal and mechanical loads. The results provide insight into the material behavior under thermo-mechanical loading and support the assessment of its suitability for the selected case study—components of air-intake systems in the automotive sector.

The novelty of this study lies in the systematic thermo-mechanical and fatigue characterization of an FFF-printed PPA CF15 composite over a temperature range of 20 to 180 °C. The results provide new insights into the temperature-dependent mechanical response of the material, including changes in stiffness, damping behavior, and fatigue performance. The experimentally obtained data serve as essential input parameters for numerical modelling and prediction of the fatigue life of lightweight structural components manufactured using AM technologies. Furthermore, the findings improve the understanding of the behavior of the material under combined thermo-mechanical loading and support the evaluation of its suitability for components of the automotive air-intake system. At the same time, the results highlight the broader potential of FFF-manufactured high-performance polymer composites for engineering applications where components operate under elevated temperatures, cyclic mechanical loads, and vibrational conditions.

## 2. Materials and Methods

### 2.1. Materials

In this study, the raw material was a thermoplastic composite filament composed of a high-temperature PPA matrix reinforced with 15% carbon fibers (“Fiberlogy”, Fiberlab S.A., Brzezie, Poland). The incorporation of carbon fibers enhances the mechanical strength and stiffness of the PPA while improving its thermal resistance and dimensional stability. Compared to unreinforced nylon materials, the composite demonstrates reduced deformation and improved wear resistance. PPA CF15 was supplied as a 1.75 mm diameter wire, the main properties of which were a density of 1.06 g/cm^3^ (ASTM D792 standard), a tensile strength of 107.724 MPa (ASTM D792 standard), and a glass transition temperature of 60.5 °C (Differential scanning calorimetry, 10 °C/min).

The composite specimens were produced using FFF technology. For this purpose, a 3D printer “Creality K1 MAX” (Creality, Shenzhen, China) was used. During the FFF process, the 3D printer followed a G-code file to deposit a thermoplastic filament layer by layer, which solidifies upon cooling to form the final structure. Since anisotropic mechanical behavior was observed in the produced specimens due to the intrinsic features of the AM process, to improve printing resolution and ensure dimensional accuracy, the main parameters of the printing process were optimized, as presented in Table 1.

**Figure 1:**
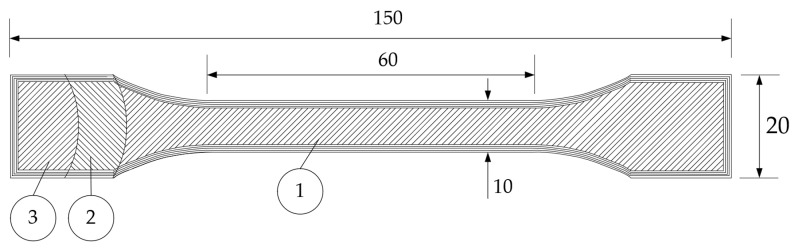
The geometry and layer orientations of the specimen: 1—top layer, 2—second layer and 3—third layer from the top surface.

The dimensions of the specimen were designed according to the ISO 527 standard [68]. The total length of the specimen was 150 mm, with a gauge length of 60 mm. The thickness of the specimen was 3 mm, while the width was measured at 10 mm in the narrow section and 20 mm in the wider section. The thickness of the printing layer was set to 0.2 mm and a raster angle of 45°/−45° was applied. The selected 45°/−45° raster angle was used to promote the distribution of load and reduce the directional anisotropy of the printed structure. All specimens were printed in horizontal orientation on a heated build platform using 100% linear infill to ensure uniform layer deposition. In addition, the use of 100% infill density helped to improve internal consolidation and reduce void formation, contributing to improved interlayer bonding and mechanical performance [69]. Nevertheless, due to the layer-by-layer nature of the FFF process, a certain degree of anisotropic behavior remains unavoidable [70]. The specimen geometry and raster angle configuration are presented in Figure 1.

PPA CF15 remains hygroscopic and environmental humidity can influence its mechanical response. To minimize moisture-related effects, the filaments were pre-dried in a convection oven at 80 °C for 12 h prior to printing. Following fabrication, the printed specimens were placed in a thermal chamber to allow temperature stabilization and to achieve uniform thermal conditions throughout the material. The chamber temperature was adjusted to match the corresponding test temperatures and the specimens were conditioned for 30 min before testing began [71]. Despite the drying procedure, the residual moisture content of the material was not measured after drying or prior to testing; therefore, it should be acknowledged that the moisture state of the PPA CF15 specimens may still affect the measured mechanical properties. All quasi-static tensile, fatigue, and DMA experiments were conducted under controlled environmental conditions, maintaining constant ambient temperature and relative humidity.

### 2.2. Methods

Mechanical characterization under quasi-static loading conditions was carried out prior to fatigue testing and numerical simulations to obtain the material parameters required for further analysis, including the elastic modulus and yield strength. Tensile experiments at elevated temperatures were performed in accordance with ISO 527 [68], following procedures commonly adopted in comparable investigations [72]. Quasi-static tests were performed using the universal testing machine “Step-Lab UD08” (STEP Engineering, Resana, Italy) equipped with an integrated thermal chamber (STEP Engineering, Resana, Italy) and the test temperature was maintained for a specified time according to the ISO 6721 standard [73]. All specimens were tested with a load rate of 1 mm/min, while the testing temperature varied between 20 °C and 180 °C. To improve the statistical consistency of the experimental data set, three independent measurements were conducted for each temperature condition. Strain values were derived from the displacement of the crosshead of the testing machine. Therefore, the deformation was assumed to be uniformly distributed along the full 110 mm distance between the grips, which included the nominal 60 mm gauge length together with adjacent transition regions. Before mechanical testing, the compliance of the testing system was determined using a reference specimen. The measured machine compliance was subsequently subtracted from the total displacement recorded during testing. Thus, the specimen deformation was calculated as the difference between the total measured displacement and the machine compliance contribution, and the elastic modulus was determined from the corrected stress–strain data.

Cyclic fatigue experiments were conducted to investigate the durability and fatigue response of the material under repeated loading conditions. Fatigue tests were performed using the universal testing machine “Step-Lab UD08” (STEP Engineering, Resana, Italy) equipped with an integrated thermal chamber (STEP Engineering, Resana, Italy). Elevated-temperature fatigue testing was carried out in accordance with the ISO 13003 standard [74]. To ensure the reproducibility and statistical consistency of the experimental results, three specimens were tested for each loading regime. The experiments were carried out under force-controlled loading using a sinusoidal waveform, while the fatigue response was evaluated under stress-controlled conditions with a load ratio of R = 0.1. A loading frequency was selected depending on the load size—4 Hz was selected for load levels up to 50% of *F_max_*, 3 Hz for 60% of *F_max_*, and 1 Hz for higher load levels in the range of 70–90% of *F_max_* to minimize self-heating effects during cyclic deformation. The applied maximum stress levels were 65.5 MPa at 20 °C and 8 MPa at 180 °C. Fatigue loading was continued either until complete specimen failure or until the predefined cycle limit was reached. The investigated temperature interval covered conditions from 20 °C to 180 °C.

DMA was conducted to characterize the viscoelastic response of the PPA CF15 material by determining the storage modulus (*E*′), the loss modulus (*E*″), and the damping factor (*tan δ*). DMA tests were performed using the universal testing machine “Step-Lab UD08” (STEP Engineering, Resana, Italy) equipped with an integrated thermal chamber (STEP Engineering, Resana, Italy). Measurements were performed in tensile mode according to the ISO 6721 standard [73]. During testing, a sinusoidal oscillatory load was applied while maintaining the strain amplitude within the linear viscoelastic range of the material. The experiments were carried out under strain-controlled multi-frequency conditions over a temperature interval from 20 °C to 180 °C, using a constant heating rate of 1 °C/min. To ensure consistency and repeatability of the obtained results, a minimum of 10 specimens were analyzed.

In addition, SEM was used to examine the surface morphology and fracture features of the specimens following tensile loading. The analysis was carried out to identify the microstructural characteristics associated with material failure, including surface imperfections, crack initiation regions, and dominant fracture mechanisms. Microstructural examination was conducted using a “FlexSEM 1000 II” microscope (Hitachi High-Tech, Tokyo, Japan). All imaging procedures were performed under high-vacuum conditions to ensure improved surface resolution and imaging quality.

## 3. Results and Discussion

### 3.1. Quasi-Static Tensile Tests

**Figure 2:**
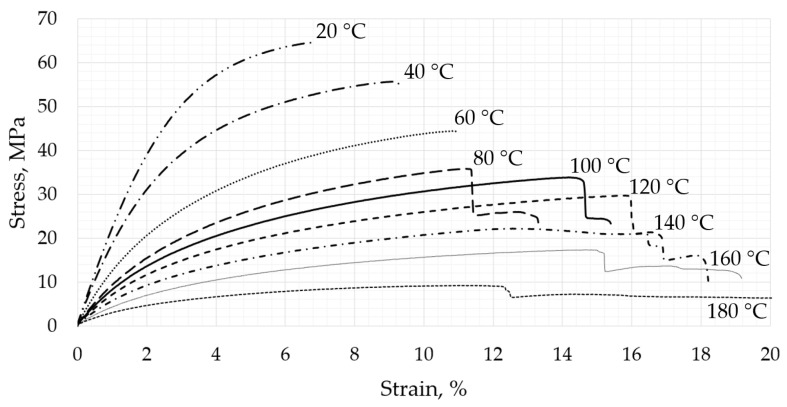
Stress–strain curves for PPA CF15 specimens.

During the quasi-static tensile tests, the main stages of the deformation process were identified: elastic deformation and nonlinear deformation. The tests conducted at different temperatures enabled the determination of the stress–strain relationships, which are presented in Figure 2.

It should be noted that the specimens analyzed in this study were fabricated using a defined set of printing conditions. The mechanical properties of polymer-based additive components can change significantly when these processing parameters are changed. Therefore, numerous studies have explored the influence of printing parameters on the mechanical properties of additively manufactured polymers, as these effects are often linked to changes in the molecular structure or fiber orientation in reinforced systems [75]. To systematically determine the relationship between the printing parameters and the mechanical properties of the resulting materials, an additional experimental study could be conducted.

Based on the experimental data obtained in this study, the main mechanical characteristics (elastic modulus, yield strength, ultimate tensile strength, tangent modulus and elongation at break) were determined. An overview of these parameters is given in Table 2.

Mechanical parameters were extracted from engineering stress–strain curves obtained during tensile tests at different temperatures. The elastic modulus was calculated from the slope of the initial linear elastic region using least-squares linear regression. The yield strength was determined according to the 0.2% offset method, where a line parallel to the elastic slope was offset by 0.2% strain and the intersection with the stress–strain curve was identified as the yield point. The ultimate tensile strength was defined as the highest stress reached during the test. The tangent modulus was determined from the slope of the stress–strain curve within the plastic region by fitting a linear function over an intermediate strain interval corresponding to 40–80% of the strain at the ultimate tensile strength. The elongation at ultimate tensile strength was taken as the strain corresponding to the maximum stress value, and the elongation at break was defined as the last recorded strain prior to failure.

The data summarized in Table 2 indicate that the elastic modulus, yield strength, and ultimate tensile strength decrease as the temperature increases, which is a characteristic response of thermoplastic materials. The reduction in these parameters reflects a loss of stiffness in the material and an increased tendency toward deformation. This behavior is mainly associated with the enhanced mobility of polymer chains at elevated temperatures. Comparable temperature-dependent reductions in stiffness and strength have been reported for PA12 and PA12-based composites, where mechanical performance at higher temperatures was predominantly governed by matrix softening effects [76,77]. In contrast, the observed increase in elongation at break suggests that the composite exhibits a transition toward more ductile behavior with increasing temperature rather than brittle failure. The observed changes in mechanical behavior are closely related to the glass transition temperature of the PPA matrix (*T_g_* = 60.5 °C). As the testing temperature approaches and exceeds *T_g_*, increased molecular mobility results in progressive softening of the polymer matrix, leading to a reduction in stiffness and strength. At the same time, the material exhibits increased ductility and altered fatigue behavior as a result of the transition from a predominantly glassy state to a more viscoelastic response. The evaluated mechanical properties were used to define the lower and up-per load limits for subsequent fatigue tests and environmental load simulations.

### 3.2. Fatigue Tests

**Figure 3:**
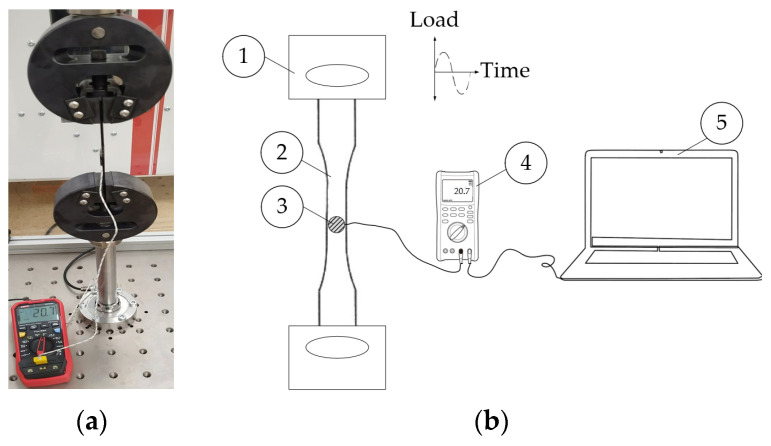
Self-heating experiment: (**a**) test equipment; (**b**) measurement principle: 1—grips for holding specimen, 2—specimen, 3—K-type thermocouple, 4—digital multimeter, 5—a laptop computer for real-time data acquisition.

Before fatigue testing, a self-heating experiment was conducted to evaluate the influence of the frequency of cyclic loading on the temperature rise of the specimens and to determine an appropriate working frequency for fatigue testing that does not induce significant self-heating effects. The specimens were subjected to sinusoidal cyclic loading in the frequency range of 1–20 Hz (1, 2, 3, 4, 5, 10, and 20 Hz) at two load levels corresponding to 50% and 70% of the maximum static load (*F_max_* = 1976 N (20 °C)). The cyclic loading was applied under tension–tension conditions with a load ratio R = 0.1. The duration of each test was 900 s. During the entire loading period, the surface temperature variation was continuously recorded using a K-type thermocouple connected to a UNI-T UT161 digital multimeter (UNI-T, Hong Kong, China), which was interfaced with a laptop computer for real-time data acquisition. The thermocouple was attached at the center of the gauge section of the specimen using Kapton tape to ensure stable thermal contact. The ambient temperature during testing was maintained at approximately 20 °C. The experimental setup and the principle of the self-heating test are presented in Figure 3.

The temporal temperature evolution during heating was modelled using Newton’s law of cooling (heating), which describes a first-order thermal response of a lumped thermal system. The evolution of temperature during heating was expressed as [78]:

*T*(*t*) = *T*_900_ − (*T*_900_ − *T*_0_) ∙ *e*^−*t*/*τ*^ (1)

where *T*(*t*) is the temperature at time *t* (°C), *T*_0_ is the initial temperature at the beginning of loading (°C), T_900_ is the temperature after 900 s (°C), *t* is the time (s) and *τ* is the thermal time constant (s).

The thermal time constant *τ* represents the characteristic response time of the system and was determined as the time required for the temperature to reach 63.2% of the total temperature increase, according to:

*T*(*τ*) = *T*_0_ + 0.632 ∙ (*T*_900_ − *T*_0_) (2)

The obtained thermal time constants demonstrated a frequency-dependent behavior, with a minimum observed in the range of 3–4 Hz at lower load levels (50% of the maximum static load). This indicates efficient heat dissipation and stable thermal conditions within this frequency range. At higher frequencies, an increase in the thermal time constant was observed, suggesting enhanced heat accumulation and possible formation of temperature gradients within the specimen. All test results are presented in Table 3.

Therefore, the fatigue testing frequency was selected within the range where both the temperature increased and the thermal time constant remained stable. The selected loading frequency was adjusted according to the applied load level to minimize self-heating effects during fatigue testing. Specifically, a loading frequency of 4 Hz was selected for load levels up to 50% of *F_max_*, 3 Hz for 60% of *F_max_*, and 1 Hz for higher load levels in the range of 70–90% of *F_max_*. Such an adaptive frequency selection approach ensures that the thermal conditions remain stable throughout the fatigue tests and prevents excessive temperature rise, which could otherwise affect the mechanical response of the polymer material and lead to premature thermal degradation.

**Figure 4:**
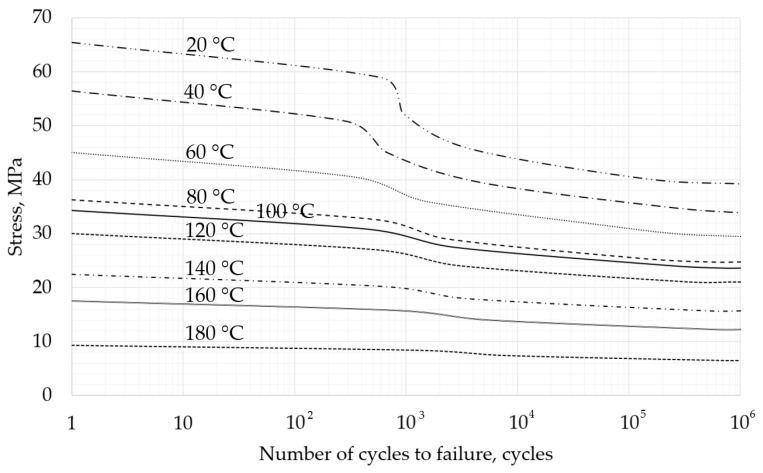
S-N curves for PPA CF15 specimens.

Fatigue tests were conducted under controlled stress amplitudes ranging from 8 MPa to 65.5 MPa at temperatures between 20 °C and 180 °C. During the experiments, the number of cycles to failure was recorded for each loading condition. Under the conditions tested, several specimens reached the run-out limit of 10^6^ cycles without failure. A summary of the fatigue test results is presented in Figure 4.

The obtained stress–number of cycles (S–N) curves indicate that the maximum load-carrying capacity decreases with increasing temperature. At high load levels, which correspond to approximately 80–90% or more of the maximum applied stress, the specimens failed after a relatively low number of cycles, regardless of the test temperature. In contrast, at lower load levels, fatigue resistance increased significantly, resulting in a substantially higher number of cycles to failure. At elevated temperatures, the mechanical response of the PPA CF15 composite exhibited increased ductility, which contributed to a reduction in crack propagation rate. This behavior is consistent with the temperature-dependent softening of the polymer matrix, which enhances energy dissipation during cyclic loading. Based on the fatigue test results, it can be concluded that the 3D-printed PPA CF15 composite is suitable for cyclic loading applications, provided that the applied stress does not exceed approximately 60–70% of the ultimate tensile strength, even under elevated temperature conditions up to 180 °C.

### 3.3. DMA Tests

**Figure 5:**
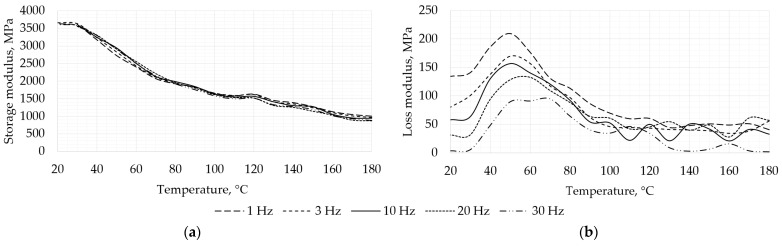
DMA results: (**a**) storage modulus; (**b**) loss modulus.

DMA was performed on PPA CF15. The storage modulus *E*′ and the loss modulus *E*″ were measured in a frequency range of 1 to 30 Hz at multiple temperatures. The measured data were subsequently used to construct frequency–temperature superposition (FTS) master curves. All calculations and data processing were performed using Microsoft Excel (Microsoft Corporation, Redmond, WA, USA). The master curves for storage modulus *E*′ and loss modulus *E*″ were obtained by horizontally shifting the frequency-dependent curves corresponding to individual temperatures along the logarithmic frequency axis. The results of the DMA test are presented in Figure 5.

The obtained results indicate that the storage modulus (*E*′) decreases progressively with increasing temperature, reflecting a reduction in material stiffness due to thermal softening of the PPA matrix. A more pronounced decrease in the storage modulus is observed in the temperature region preceding the *T_g_*, while beyond this region, the modulus decreases more gradually. This behavior can be attributed to the reinforcing effect of carbon fibers, which contributes to an increase in stiffness at lower temperatures. However, the reinforcing efficiency of the fibers diminishes at elevated temperatures as the mobility of the polymer matrix increases. Consequently, a noticeable change in the slope of the modulus curve is observed after passing the glass transition region. The loss modulus (*E*″) exhibits a significant increase within the glass transition region, indicating enhanced molecular mobility and increased energy dissipation within the polymer matrix. Furthermore, analysis of frequency-dependent behavior indicates that at excitation frequencies above approximately 20 Hz, the applied loading frequency exceeds the molecular relaxation rate, resulting in reduced energy dissipation within the system.

In order to extend the applicability of the experimentally obtained DMA results beyond the measured frequency range, FTS was applied to construct master curves of the storage modulus *E*′ and the loss modulus *E*″. The experimental measurements were limited to frequencies up to 30 Hz; however, the subsequent stages of this research involve finite element analysis (FEA) of the dynamic response of PPA CF structures subjected to mechanical vibrations. In these simulations, excitation frequencies exceeding 500 Hz are expected, which are significantly higher than the experimentally accessible range. Therefore, the viscoelastic behavior of the material at higher frequencies was estimated by extrapolating the measured data using analytically fitted master curve functions. This approach enables the prediction of storage and loss modulus values at elevated frequencies required for numerical modelling while maintaining consistency with the experimentally determined viscoelastic response of the PPA CF composite. The master curve of the storage modulus was approximated using a hyperbolic tangent function [79]:

*E*′(*f*) = *a* ∙ *tanh* (*b* (*log*_10_
*f* + *c*)) + *d* (3)

where *a*, *b*, *c* and *d* are the fit coefficients.

The loss modulus was derived from the analytical derivative of the storage modulus function:

*E*″(*f*) = *πab*/2 ∙ *sech*^2^ (*b* (*log*_10_
*f* + *c*)) (4)

Parameters *a*, *b*, *c*, and *d* were determined by nonlinear regression using iterative optimization in Microsoft Excel (Microsoft Corporation, Redmond, WA, USA). The optimization was performed by minimizing the squared difference between experimental and modeled values of both the storage and loss moduli. Following extrapolation, the fitting function coefficients were determined together with the corresponding goodness-of-fit parameter (*R*^2^). The fit function coefficients were calculated at selected temperature values of 80 °C, 100 °C and 120 °C, because in further studies the operating temperature of the composite application does not exceed 100 °C. The resulting coefficients are summarized in Table 4.

**Figure 6:**
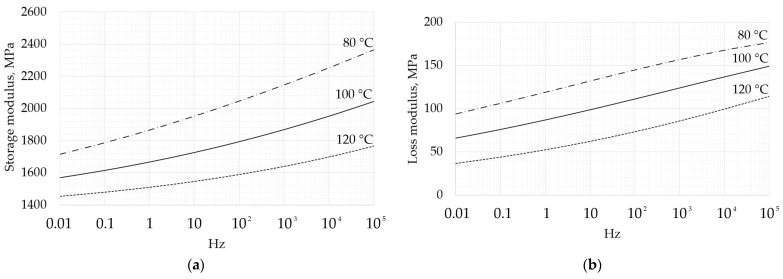
Master curves of PPA CF15 composite: (**a**) storage modulus; (**b**) loss modulus.

After determining the coefficients, they were substituted into the corresponding equation, allowing the construction of the master curves for both the storage and the loss modulus. The frequency range was selected from 0.001 Hz to 10^5^ Hz due to its wider engineering applicability. The resulting master curves for storage and loss moduli are presented in Figure 6.

Figure 6 presents the storage and loss moduli as frequency functions for PPA CF15 at selected temperatures (80 °C, 100 °C, and 120 °C). Both moduli increased with frequency, reflecting the rate-dependent viscoelastic nature of the polymer composite. The results indicate that the material becomes progressively stiffer and exhibits increased energy dissipation at higher loading frequencies. The observed behavior is consistent with typical viscoelastic responses reported for reinforced PPA-based composites.

### 3.4. SEM Analysis

**Figure 7:**
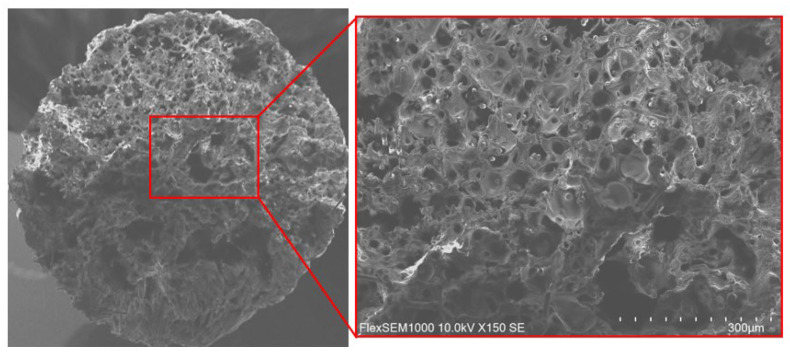
Cross-sectional view of a PPA CF15 filament.

SEM analysis was performed to evaluate the microstructure of the PPA CF15 filament and the fracture surfaces formed after tensile tests at different temperatures. In Figure 7, the SEM images represent the cross-sectional view of the PPA CF15 filament. The filament structure exhibits a heterogeneous porous morphology, characterized by pores and voids of varying sizes. This porosity is likely associated with air entrapment occurring during the extrusion process. In addition, carbon fiber fragments embedded within the polymer matrix are clearly visible, confirming the composite nature of the material.

**Figure 8:**
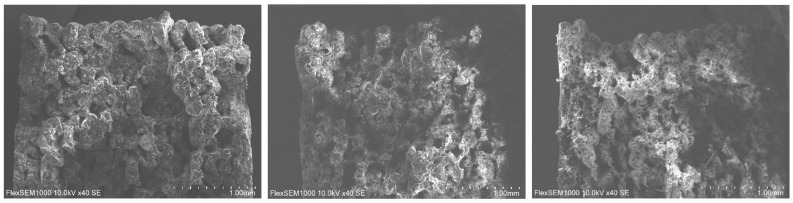

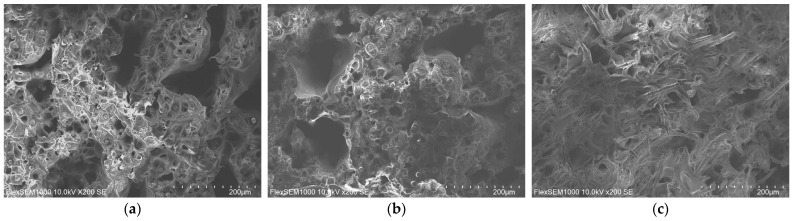
Cross-sectional view of PPA CF15 specimens after deformation at different temperatures: (**a**) 20 °C; (**b**) 60 °C; (**c**) 120 °C.

The SEM images presented in Figure 8 illustrate the fracture surfaces after tensile testing at 20 °C, 60 °C, and 120 °C. With increasing temperature, a noticeable increase in plastic deformation characteristics and fiber pull-out phenomena was observed. These microstructural observations correlate well with the results of the tensile test, which demonstrated a decrease in strength and a greater increase in deformation with increasing temperature.

At 20 °C, the fracture surface exhibits an irregular and rough morphology with distinct features typical of a brittle fracture. Microcrack lines and fiber pull-out traces are visible, indicating partial debonding of carbon fibers from the polymer matrix. Opened pore structures are also observed, which may act as stress concentration sites during loading. This morphology suggests that at lower temperatures, the material exhibits higher stiffness but limited ductility.

When the temperature increases to 60 °C, more pronounced plastic deformation behavior is observed. The fracture surface becomes less brittle and elongated matrix structures together with plastically deformed regions become visible. Longer fiber pull-out channels are evident, indicating reduced matrix stiffness and improved deformation capability prior to fracture. These observations correlate with the experimentally observed increase in fracture strain and decrease in tensile strength.

At 120 °C, the fracture surface demonstrates significantly enhanced plastic deformation features. Stretched and merged matrix structures are visible, together with larger deformed regions, indicating substantial softening of the material. The fiber–matrix interaction appears weaker, and fiber pull-out events become more frequent. This observation suggests that at elevated temperatures, the polymer matrix loses a portion of its mechanical resistance, leading to fracture through a plastically deformed matrix rather than brittle failure.

These observations confirm that the temperature-dependent mechanical response of the PPA CF15 composite is strongly governed by matrix softening and fiber–matrix interfacial behavior, which significantly influences the overall fracture mechanism.

## 4. Case Study Analysis

### 4.1. Dynamic and Thermal System Analysis

The case study was conducted to identify the behavior of the PPA CF15 composite under real operating conditions involving simultaneous dynamic, pressure, and thermal loading. Experimental investigations were performed using a Volkswagen gasoline turbocharged AWT 110 kW engine (Volkswagen, Wolfsburg, Germany). Resonance frequencies, acceleration amplitudes, operating pressure, and temperatures of the engine block and intake air were determined. The obtained values were subsequently used as input parameters for the FEA.

**Figure 9:**
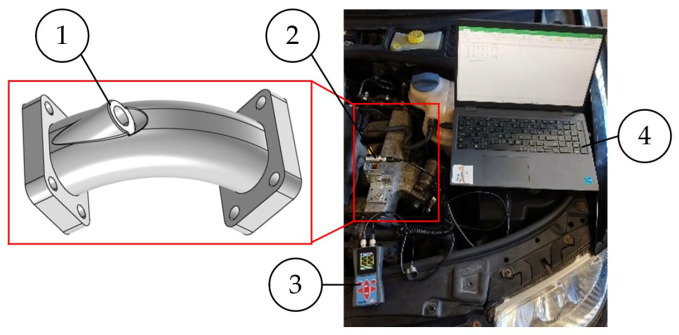
Experimental setup for vibration testing: 1—segment of the air-intake manifold; 2—a sensor connected to the engine; 3—a VM25 vibration meter; 4—a computer.

Initially, the vibration behavior of the engine was evaluated through vibration measurements. Resonance frequencies and acceleration values were determined to calculate the forces generated due to vibrational loading. Additional components were mounted in the IM, including a throttle valve with a mass of 0.93 kg and an intake distributor with a mass of 1.02 kg. Vibrations were recorded in three orthogonal directions (X, Y, and Z) using a VM25 vibration meter (Metra Meß- und Frequenztechnik Radebeul GmbH & Co. KG, Radebeul, Germany). The experimental setup is presented in Figure 9 and the obtained vibration results are summarized in Table 5.

**Figure 10:**
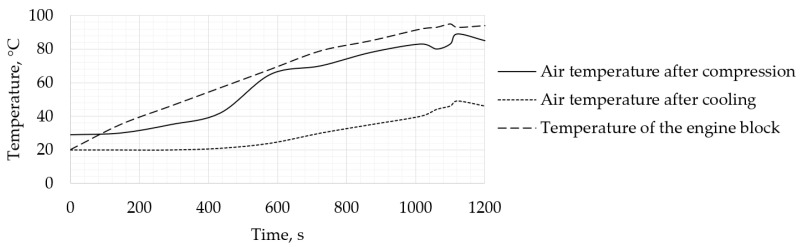
Temperature distribution at different air intake zones.

To fully define the mathematical model, temperature variations that affect the IM were also monitored. The IM mounting interface was influenced by the engine block temperature. The internal manifold walls were exposed to intake air heated during turbocharger compression, while the external surface of the manifold was subjected to ambient temperature conditions. In addition, the intake air temperature was recorded before and after the intercooler. The measured temperature variations are presented in Figure 10.

Finally, due to the operation of the turbocharger, a maximum pressure of 133 kPa enters the IM, while the ambient pressure was 103 kPa. Experimentally determined resonant frequencies, acceleration amplitudes, pressure data, and temperature values were the main input parameters for the FEA. Based on these data, a realistic numerical model of the IM was created, allowing simulation of coupled thermomechanical and dynamic loading conditions.

### 4.2. Finite Element Analysis

Engineering components operating under elevated temperatures and dynamic loads are commonly encountered in practical applications. In this study, numerical simulations were performed to evaluate whether the PPA CF15 composite is suitable for such operating conditions. For this purpose, a gasoline internal combustion engine equipped with a turbocharger was selected as the reference system. In this configuration, the IM is subjected not only to vibration-induced forces but also to internal pressure loads.

FEA was carried out using COMSOL Multiphysics^®^ 6.4 (COMSOL AB, Stockholm, Sweden). The developed model incorporates experimental material data obtained from tensile and DMA tests, as well as operational parameters measured under various engine regimes, including resonance frequencies, vibration acceleration amplitudes, operating temperatures, and internal pressures. The IM geometry was simplified to represent only the critical region prone to stress concentration. The geometry of the model was developed based on actual IM dimensions using SolidWorks 2025 (Dassault Systèmes SolidWorks Corp., Waltham, MA, USA). Boundary conditions were specified to accurately represent real mounting conditions, with the manifold rigidly fixed to the engine block. The internal surface of the IM was subjected to a boundary pressure, representing the turbocharger-generated pressure of 133 kPa. The upper section of the manifold was loaded using a boundary force that represents the mass of the attached intake components, including the throttle body (0.93 kg) and the intake distributor (1.02 kg). Under these loading conditions, the maximum force acting on the IM occurs at 4000 RPM, corresponding to the maximum acceleration amplitude. The forces acting in three spatial directions were calculated using Newton’s second law and experimentally measured acceleration data:

*F_cycX_* = 1.95 ∙ 139 ∙ *sin* (2*π* ∙ 127 ∙ *t*) (5)

*F_cycY_* = 1.95 ∙ 258 ∙ *sin* (2*π* ∙ 133 ∙ *t*) (6)

*F_cycZ_* = 1.95 ∙ 9.822 + 1.95 ∙ 80 ∙ *sin* (2*π* ∙ 133 ∙ *t*) (7)

**Figure 11:**
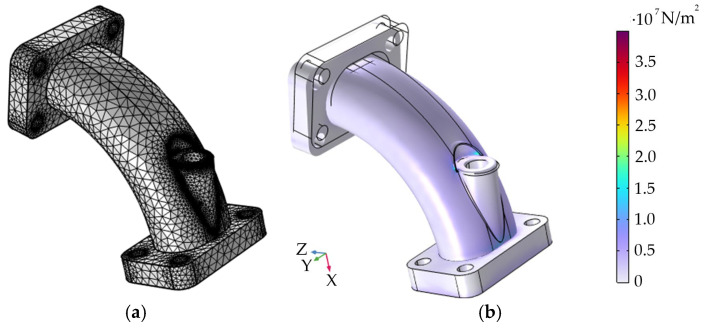
Finite element analysis: (**a**) Meshing of the mathematical model; (**b**) Von Mises stress distribution.

Because the component was to be produced using AM, the material was considered non-homogeneous. The raster angle was therefore aligned with the direction of the highest expected stresses. Although this approach does not explicitly account for fiber orientation, fiber–matrix interfacial effects, or internal defects, it provides a computationally efficient and sufficiently accurate representation of the global structure. Although these factors may influence local stress concentrations and anisotropic material behavior, their inclusion would significantly increase the complexity of the model and computational cost. Therefore, the obtained numerical results should be regarded as an engineering approximation of the thermo-mechanical performance of the IM. The mathematical model of the IM is presented in Figure 11.

The FEA performed allowed for evaluation of the suitability of both the manifold geometry and the selected material under cyclic dynamic loading at elevated temperatures. The results of the static analysis indicated that the highest levels of deformation occurred in the mounting regions and at the transitions between the flange and the air channel, where the effects of stress concentration were the most pronounced. The maximum deformation values remained below the critical limits of the PPA CF15 composite, as determined experimentally during the material testing.

To verify numerical reliability, a mesh independence study was performed. The results demonstrated that further mesh refinement resulted in only minor variations in the measured values, confirming mesh convergence. To maintain a balance between computational efficiency and solution accuracy, the Finer mesh configuration was selected for further simulations. The mesh convergence results are summarized in Table 6.

**Figure 12:**
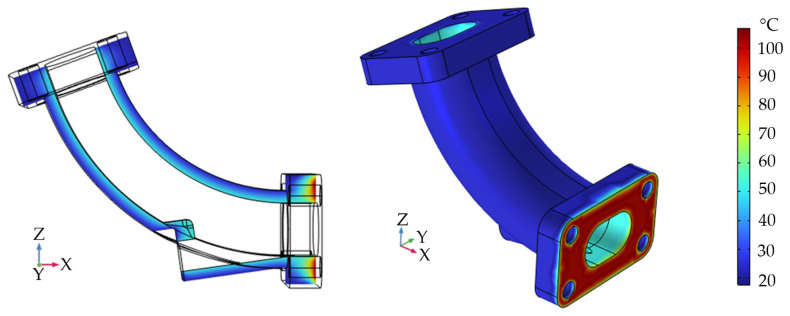
The resulting intermediate temperature distribution across the manifold, corresponding to three thermal zones.

To evaluate the geometry, recalculated loading forces were applied together with thermal boundary conditions. The IM was subjected to multiple temperature regions that represent realistic engine operation. The engine block temperature was assumed to reach a maximum of 95 °C, and this temperature was applied at the fixed constraint location. The external surface of the manifold was exposed to ambient air conditions, represented by a temperature of 22 °C. The internal surfaces were exposed to compressed air that exited the turbocharger, with a maximum temperature of 49 °C. To account for the combined influence of mechanical and thermal loads, a Multiphysics simulation was implemented. The resulting intermediate temperature distribution across the manifold, corresponding to three thermal zones, is presented in Figure 12.

To evaluate the viscoelastic response of the material, a time-dependent simulation was performed. The evolution of the first principal strain, displacement magnitude and von Mises stress was monitored over a simulation period of 10^4^ s and summarized in Table 7.

The results indicate a gradual increase in deformation during the initial loading stage, followed by stabilization at longer times. Specifically, the first principal strain increased from 20.042 × 10^−3^ in the initial stage to 25.656 × 10^−3^ in 10^2^ s, after which no significant changes were observed up to 10^3^ s. A similar trend was observed for the displacement magnitude, which increased from 1.056 × 10^−3^ m to 1.401 × 10^−3^ m, indicating a time-dependent deformation typical of viscoelastic creep behavior. The von Mises stress decreased from 8.95 × 10^7^ Pa to approximately 7.20 × 10^7^ Pa, followed by minor fluctuations and eventual stabilization. This behavior indicates stress relaxation and redistribution within the component.

Overall, the obtained results demonstrate a clear time-dependent mechanical response, confirming that the inclusion of viscoelastic material properties derived from the DMA master curve data enables a realistic prediction of the evolution of the deformation under combined thermal and mechanical loading conditions. The stabilization of deformation observed after approximately 10^2^ s suggests that the majority of viscoelastic deformation occurs within the initial loading phase, after which only negligible additional deformation is expected. This finding is particularly important for evaluating the long-term structural reliability of IM under sustained thermal and mechanical loads.

## 5. Conclusions

This study investigated the thermo-mechanical performance of a 3D-printed PPA CF15 composite manufactured using FFF. The material behavior was evaluated through quasi-static tensile testing, fatigue testing, DMA, SEM, and FEA under elevated temperature conditions representative of automotive air-intake system operation.

The quasi-static tensile tests demonstrated a clear temperature-dependent reduction in mechanical properties. The elastic modulus decreased from 2.437 GPa at 20 °C to 0.401 GPa at 180 °C, while the ultimate tensile strength decreased from 64.537 MPa to 9.190 MPa in the same temperature range. In contrast, elongation at break generally increased with temperature, indicating a transition from relatively brittle behavior at lower temperatures to more ductile behavior at elevated temperatures. These results confirm that the mechanical response of PPA CF15 is strongly governed by thermal softening of the polymer matrix.

Fatigue testing revealed that the fatigue resistance of the PPA CF15 composite decreases with increasing temperature and applied stress levels. At stress amplitudes exceeding approximately 80–90% of the maximum applied stress, rapid failure occurred regardless of temperature. However, at lower stress levels, significantly longer fatigue lives were achieved, and several specimens reached the run-out limit of 10^6^ cycles. The results indicate that reliable cyclic performance can be achieved when the applied stress remains below approximately 60–70% of the ultimate tensile strength, even at elevated temperatures up to 180 °C. The implementation of adaptive frequency selection based on self-heating analysis ensured stable thermal conditions during fatigue testing.

DMA confirmed the viscoelastic nature of the PPA CF15 composite. The storage modulus decreased with increasing temperature, while both the storage modulus and the loss modulus increased with loading frequency, reflecting rate-dependent material behavior. The application of frequency–temperature superposition enabled the construction of master curves that extended material characterization to frequencies up to 10^5^ Hz. The developed analytical functions demonstrated good agreement with the experimental data, enabling reliable prediction of the viscoelastic properties required for numerical simulations.

SEM observations revealed significant microstructural changes associated with an increase in temperature. At lower temperatures, the fracture surfaces exhibited brittle characteristics with visible microcracks and limited plastic deformation. With increasing temperature, enhanced matrix deformation and pronounced fiber pull-out were observed, indicating a progressive weakening of the fiber-matrix interfacial bonding. These observations correlate well with the experimentally measured reduction in stiffness and strength.

Finite element simulations incorporating experimentally derived mechanical and viscoelastic properties demonstrated that the selected PPA CF15 composite is capable of sustaining combined thermal and mechanical loading conditions representative of turbocharged engine IM operation. The results indicated that the deformation and stress levels remained below the critical limits and viscoelastic stabilization occurred after approximately 10^2^ s of loading, suggesting acceptable long-term structural performance.

Overall, the obtained results confirm that additively manufactured PPA CF15 composites exhibit promising thermo-mechanical performance suitable for lightweight structural components operating under elevated temperature and cyclic loading conditions. The integration of experimental characterization and numerical modelling provides a comprehensive framework for predicting material behavior in engineering applications. The findings support the potential application of FFF-manufactured PPA CF15 composites in automotive air-intake systems and similar environments that require a high strength-to-weight ratio, thermal stability, and fatigue resistance.

## Figures and Tables

**Table 1:** The printing parameters used for fabrication of PPA CF15 specimens.

Parameters		
Structure	Raster angle	45°
	Infill density	100%
	Layer thickness	0.2 mm
	The number of contours	5
	Diameter of the nozzle	0.6 mm
	Weight of the specimen	6.13 g
Temperature	Nozzle temperature	290 °C
	Platform temperature	100 °C
	Chamber temperature	50 °C
Speed	Printing speed	80 mm/s
	Extrusion speed	80 mm/s

**Table 2:** Mechanical parameters of PPA CF15.

Temperature, °C	Elastic Modulus, GPa	Yield Strength, MPa	Ultimate Tensile Strength, MPa	Tangent Modulus, GPa	Elongation at Break, %
20	2.437 ± 0.19	30.591 ± 1.12	64.537 ± 0.88	0.533 ± 0.0032	6.750 ± 1.09
40	2.166 ± 0.17	21.332 ± 1.12	55.719 ± 0.91	0.287 ± 0.0030	9.084 ± 1.20
60	1.489 ± 0.16	13.583 ± 1.22	44.382 ± 0.81	0.230 ± 0.0029	10.806 ± 1.09
80	1.107 ± 0.12	10.175 ± 1.30	35.791 ± 0.98	0.191 ± 0.0018	11.195 ± 1.13
100	0.977 ± 0.09	9.052 ± 1.35	33.841 ± 1.11	0.132 ± 0.0022	14.167 ± 1.23
120	0.857 ± 0.05	7.580 ± 1.29	29.641 ± 1.03	0.101 ± 0.0026	15.806 ± 1.52
140	0.705 ± 0.06	5.855 ± 1.36	22.193 ± 0.98	0.086 ± 0.0018	12.556 ± 1.88
160	0.530 ± 0.02	4.372 ± 1.42	17.275 ± 0.88	0.064 ± 0.0009	14.667 ± 2.08
180	0.401 ± 0.03	2.803 ± 1.50	9.190 ± 0.76	0.041 ± 0.0008	11.472 ± 1.99

**Table 3:** Results of self-heating experiment at different frequencies.

50% of the Maximum Static Load					70% of the Maximum Static Load				
Frequency, Hz	Heat_min_, °C	Heat_max_, °C	Heat_0.632_, °C	*τ*, s	Frequency, Hz	Heat_min_, °C	Heat_max_, °C	Heat_0.632_, °C	*τ*, s
1	19.8	20.6	20.3056	300	1	20.0	21.4	20.8848	243
2	20.2	21.3	20.8952	256	2	20.8	23.8	22.6960	256
3	20.2	21.9	21.2744	246	3	20.7	25.4	23.6704	297
4	20.3	22.6	21.7536	245	4	20.9	28.5	25.7032	350
5	20.4	23.2	22.1696	254	5	21.0	31.2	27.4464	361
10	20.5	26.8	24.4816	300	10	-	-	-	-
20	-	-	-	-	20	-	-	-	-

**Table 4:** Calculated fit function coefficients with the goodness-of-fit parameter.

Temperature, °C	*a*	*b*	*c*	*d*	*R* ^2^
80	1500	0.0800	−7.842	2700	0.97
100	1320	0.0897	−10.330	2629	0.96
120	1300	0.1000	−13.000	2629	0.98

**Table 5:** Data on resonant frequency and acceleration of the gasoline engine.

Revolutions Per Minute (RPM)	X Direction		Y Direction		Z Direction	
	Resonant Frequency, Hz	Acceleration, m/s^2^	Resonant Frequency, Hz	Acceleration, m/s^2^	Resonant Frequency, Hz	Acceleration, m/s^2^
800	23	1.4	139	0.8	23	1.1
1000	185	0.9	470	1.3	28	1.0
1500	52	1.0	52	1.0	52	5.0
2000	63	4.0	63	2.0	63	8.0
2500	81	9.0	81	3.0	75	11.0
3000	98	16.0	98	4.0	98	18.0
3500	231	35.0	556	6.0	110	30.0
4000	127	139.0	133	258.0	133	80.0

**Table 6:** The mesh reliability analysis.

	Extra Fine	Finer	Fine	Normal	Coarse	Coarser
The number of domain elements	23.0865 × 10^4^	9.5010 × 10^4^	3.6977 × 10^4^	1.7276 × 10^4^	0.8327 × 10^4^	0.4939 × 10^4^
The number of boundary elements	3.9984 × 10^4^	2.1582 × 10^4^	1.1528 × 10^4^	0.6804 × 10^4^	0.4142 × 10^4^	0.2840 × 10^4^
Value of the first principal strain	8.9901 × 10^−3^	8.9831 × 10^−3^	8.5893 × 10^−3^	7.5893 × 10^−3^	6.6491 × 10^−3^	5.6912 × 10^−3^
Displacement magnitude, m	4.9076 × 10^−4^	4.9075 × 10^−4^	4.9055 × 10^−4^	4.9035 × 10^−4^	4.8656 × 10^−4^	4.7647 × 10^−4^

**Table 7:** Time-dependent simulation results.

Time, s	First Principal Strain	Displacement Magnitude, m	Von Mises Stress, N/m^2^
0.01	20.042 × 10^−3^	1.056 × 10^−3^	8.95 × 10^7^
0.1	21.306 × 10^−3^	1.134 × 10^−3^	7.13 × 10^7^
1	21.86 × 10^−3^	1.170 × 10^−3^	6.89 × 10^7^
10	23.937 × 10^−3^	1.312 × 10^−3^	7.06 × 10^7^
10^2^	25.641 × 10^−3^	1.400 × 10^−3^	7.32 × 10^7^
10^3^	25.656 × 10^−3^	1.401 × 10^−3^	7.21 × 10^7^
10^4^	25.656 × 10^−3^	1.401 × 10^−3^	7.19 × 10^7^

## Data Availability

The original contributions presented in this study are included in the article. Further inquiries can be directed to the corresponding authors.
